# Burnout Assessment Tool (BAT): Validity Evidence from Brazil and Portugal

**DOI:** 10.3390/ijerph19031344

**Published:** 2022-01-25

**Authors:** Jorge Sinval, Ana Claudia S. Vazquez, Claudio Simon Hutz, Wilmar B. Schaufeli, Sílvia Silva

**Affiliations:** 1 Business Research Unit (BRU-IUL), Instituto Universitário de Lisboa (ISCTE-IUL), 1649-026 Lisbon, Portugal; silvia.silva@iscte-iul.pt; 2 William James Center for Research, ISPA—Instituto Universitário, 1149-041 Lisbon, Portugal; 3 Faculty of Philosophy, Sciences and Languages of Ribeirão Preto, University of São Paulo, Ribeirão Preto 14040-901, SP, Brazil; 4 Faculdade de Medicina, Universidade de Lisboa, 1649-028 Lisbon, Portugal; 5 Department of Psychology, Universidade Federal de Ciências da Saúde de Porto Alegre, Porto Alegre 90050-170, RS, Brazil; anasv@ufcspa.edu.br; 6 Department of Psychology, Universidade Federal do Rio Grande do Sul, Porto Alegre 90035-002, RS, Brazil; claudio.hutz@gmail.com; 7 Research Unit Occupational & Organizational Psychology and Professional Learning KU Leuven, 3000 Leuven, Belgium; wilmar.schaufeli@kuleuven.be; 8 Department of Psychology, Utrecht University, 3584 CS Utrecht, The Netherlands

**Keywords:** Brazil, burnout, Burnout Assessment Tool (BAT), cross-cultural adaptation, measurement invariance, multi-occupational, Portugal, psychometrics, Rasch analysis, validity evidence

## Abstract

The Burnout Assessment Tool (BAT) has been gaining increased attention as a sound and innovative instrument in its conceptualization of burnout. BAT has been adapted for several countries, revealing promising validity evidence. This paper aims to present the psychometric properties of the Brazilian and Portuguese versions of the BAT in both the 23-item and 12-item versions. BAT’s validity evidence based on the internal structure (dimensionality, reliability, and measurement invariance) and validity evidence based on the relations to other variables are the focus of research. A cross-sectional study was conducted with two non-probabilistic convenience samples from two countries (*N* = 3103) one from Brazil (*n_Brazil_* = 2217) and one from Portugal (*n_Portugal_* = 886). BAT’s original structure was confirmed, and it achieved measurement invariance across countries. Using both classic test theory and item response theory as frameworks, the BAT presented good validity evidence based on the internal structure. Furthermore, the BAT showed good convergent evidence (i.e., work engagement, co-worker support, role clarity, work overload, and negative change). In conclusion, the psychometric properties of the BAT make this freely available instrument a promising way to measure and compare burnout levels of Portuguese and Brazilian workers.

## 1. Introduction

Although the burnout syndrome appeared in the 1970s, it is still a global issue such that the 11th revision of the International Classification of Diseases of World Health Organization (ICD-11) defines it as an occupational phenomenon with risk of harming health [1]. The adopted definition of burnout in the ICD-11 comprises three factors (exhaustion, cynicism, and reduced professional efficacy) as the framework proposed by Maslach et al. [2]. However, the conceptualization of burnout is somewhat controversial [3]; for example, a meta-analytical study on the physicians’ burnout found 142 unique definitions of burnout with at least 47 unique definitions using MBI. Some constructs, such as depression and fatigue, are conceptually linked to job burnout [4,5]. These phenomena are potentially part of the process of long-term sick leave. At the core of burnout lies severe fatigue (i.e., exhaustion); however persistently fatigued workers are not necessarily (by definition) in burnout, nor must burned-out workers necessarily report fatigue as the main complaint [5]. Occupational fatigue has been linked to an imbalance between the intensity and duration and timing of work with recovery time [6]. Studies over decades have shown evidence that burnout syndrome predicts various negative consequences to individuals and organizations, such as cardiovascular diseases, hypercholesterolemia, type 2 diabetes, coronary heart disease, musculoskeletal disorders, prolonged fatigue, headaches, gastrointestinal issues, mood disturbance, depressive symptoms, absenteeism, poor performance, insomnia, depressive symptoms, and life and job dissatisfaction [7,8,9,10,11,12,13,14,15,16].

Research shows that job demands (e.g., work overload) are more associated with job burnout, while job resources (e.g., co-worker support) are more related to job burnout’s antipode, i.e., work engagement [17]. Nowadays, researchers claim the COVID-19 pandemic has posed strain and increased workload and job stress, particularly in healthcare workers, who have presented a higher risk of burnout than other occupations [18,19,20]. Going beyond the individual consequences of burnout, recent research has also investigated burnout in a large range of occupations, organizations, and countries [21,22,23,24,25,26]. The literature has firmly established that burnout is not only detrimental for workers’ health but also has negative effects at the organizational level.

The most widely used instrument to assess burnout is the Maslach Burnout Inventory (MBI) [27]. Despite MBI’s early contribution to enlighten burnout as an important psychological state to be deeply studied, researchers are still discussing its theoretical framework and psychometric basis and the practical applicability of this instrument [21,28]. Schaufeli et al. [29] summarize these criticisms of MBI as including the following as the most important: (a) the questioning of the validity evidence of the constituting dimensions of burnout, (b) the lack of clinically established cut-off values, (c) the lack of representative and national samples to ground its statistical norms, (d) the limitations of its practical usability, and (e) the inconsistent dimensionality also in the cross-national studies on MBI [30]. Finally, with similar problems and weaknesses, there are other burnout measures, such as the Copenhagen Burnout Inventory [31], the Oldenburg Burnout Inventory [32], and, recently, the COVID-19 Burnout Scale [33].

### 1.1. Burnout Assessment Tool (BAT)

Taken together, these criticisms call for an alternative instrument to assess burnout and overcome these flaws using a novel conceptualization of the matter, which has been addressed by the development of the Burnout Assessment Tool (BAT) [34]. The BAT instrument assumes that burnout is a syndrome assessed by core symptoms (exhaustion, mental distance, emotional impairment, and cognitive impairment) and secondary symptoms (psychological distress and psychosomatic complaints), which could be associated with depressed mood and other comorbidities. Therefore, BAT considers burnout a second-order factor that acts as a syndrome, meaning that all four components are connected and belong to the same higher-order construct, i.e., burnout [21]. Based on the Job Demands–Resources Model (JD-R) [17], the key components constituting the burnout process are the draining energy that leads to feeling exhausted and extremely tired at the same time that the distancing mentally manifests itself as a lack of interest and aversion to work [35]. In addition, in-depth interviews with experts brought two significant dimensions of burnout, which were not known until then, which are emotional impairment and cognitive impairment. These dimensions affect one’s self-regulation to deal adequately with the daily working activities and to recover self-energy linked to the motivational process [36].

Meanwhile, BAT has largely been investigated [37,38,39,40,41] and has demonstrated measurement invariance between seven countries in Europe and Japan [21]. As in the works of De Beer et al. [21] and Sakakibara et al. [28], the current study is based on the BAT reconceptualization of burnout as a work-related state of exhaustion, extreme tiredness with reduced ability to regulate cognitive and emotional processes, and mental distancing. It can develop depressed mood as well as non-specific psychological and psychosomatic complaints [34]. Despite using the raw scores of only one item from MBI (i.e., “I feel exhausted at the end of the working day”), Schaufeli [24] found medium levels of burnout in Portugal, in comparison with a random sample of workers from thirty-five European countries (*n* = 43,675), using data from the 6th European Working Conditions Survey [42]. While in Brazil there is no publication reporting burnout scores using a survey (conducted with a representative sample) at the national level.

This current study is focused on the psychometric properties of BAT from a cross-national perspective (i.e., Brazil and Portugal). The main goal is to assess BAT’s validity evidence based on the internal structure and based on the relations to other variables.

### 1.2. Research Hypotheses

Following the recommendations of the Standards for Educational and Psychological Testing [43], this study aims to evaluate two types of validity evidence for both for BAT’s Portuguese and Brazilian version: one related to the internal structure, and one based on the relations to other variables (i.e., work engagement, role clarity, co-worker support, work overload, and negative change). BAT’s original structure was successfully confirmed in several countries in a study by De Beer et al. [21] with data from Austria, Belgium (Flanders), Finland, Germany, Ireland, Japan, and the Netherlands. The Japanese version of BAT was also confirmed in a different study [28], while the South Korean version maintained the hierarchical structure with four first-order dimensions, albeit with the removal of one item from the mental distance factor [44]. The Russian version also provided evidence indicative of the stability of the hierarchical structure [45]. Altogether, it is expected that the hypothesized hierarchical structure for BAT-23 (one second-order latent variable with four first-order factors, 23 indicators) and BAT-12 (second-order factor with four first-order dimensions, 12 items) hold with a satisfactory fit to the data in both Brazil and Portugal (H1). The reliability of the scores is one of the key components of the internal structure of any psychometric instrument [46]. It can be analyzed using four different types of approaches: internal consistency, test–retest, parallel forms, and interrater agreement. Previous research showed good evidence of internal consistency estimates using the ordinal α [47] for both second-order factor and first-order dimensions [21]. BAT’s also presented satisfactory evidence in terms of test–retest evidence [48]. The second hypothesis (H2) states that BAT presents satisfactory internal consistency estimates (≥0.80) [46]. Measurement invariance is another component of the validity evidence based on the internal structure; it is an essential feature that is necessary before any substantive group comparisons (e.g., countries, sex) can be established. BAT has shown measurement invariance between seven countries Austria, Belgium (Flanders), Finland, Germany, Ireland, Japan, and the Netherlands [21]. Regarding sex, there is no single study investigating measurement invariance among sex. However, it is known that sex might be an important factor regarding burnout [49], it is expected that females present higher levels of burnout [50] namely in terms of exhaustion [51,52]. While others did not reach definite conclusions [25].

Other instruments measuring burnout and related constructs have previously shown measurement invariance among workers from Brazil and Portugal [53] and among sex within the two mentioned countries [54,55]. Reinforcing the similarity of the measurement structure of psychometric instruments among workers from the two countries. It is hypothesized (H3) that BAT holds measurement invariance among countries (Brazil and Portugal), and sex.

Another important source of validity evidence is provided by the relationship of instrument scores to external variables to the instrument [43]. This source of evidence allows understanding if the interpretation of the scores can be done as expected by the nomological network of constructs [56]. The JD-R model identifies possible antecedents of job burnout [57]. The central idea of the JD-R model is that working conditions, which are specific to every occupation, can generally be classified as either job demands or job resources, and those job characteristics will contribute to job burnout and work engagement [58]. The JD-R model suggests that work engagement is negatively related to burnout, since high job demands lead to a health impairment process (i.e., job burnout) and high resources will lead to a motivational process, i.e., work engagement [59]. Several meta-analyses have supported the relationship between job demands and resources and burnout [60,61,62]. In these studies, several job demands and resources were identified. For instance, social support, workload, and role clarity have been found as relevant demands and resources. As such, it is expected to observe a positive association between burnout and job demands and a negative relation between burnout and job resources [28,39,63]. It is anticipated that BAT’s scores are negatively correlated with work engagement, role clarity, and co-worker support and positively correlated with work overload, and negative change (H4).

## 2. Methods

### 2.1. Sampling, and Data Collection

In this cross-sectional survey, a non-probabilistic convenience sample was collected. The inclusion criteria consisted of participants being able to read Portuguese and having easy access to a smartphone, PC, or tablet where they could open a digital questionnaire. The authors invited workers from Brazil and Portugal to participate. Considering BAT’s second-order factor and four first-order dimensions with 23 manifest variables, it results in a total of 226 degrees of freedom [64], assuming that the population RMSEA should be not higher than 0.08 (i.e., ε₀ = 0.08; *H*_0_: ε ≥ 0.08), since rejecting this hypothesis will lead to the conclusion that the model fit is better than 0.08, the recommended cutoff for a reasonable fit [65]. Additionally, the true population RMSEA was considered to be ε = 0.064 based on the findings from de Beer’s et al. [21] study using a sample of 10,138 participants. Altogether, for an α = 0.05, β = 0.20 (i.e., power = 0.80) resulted in a required sample size of *n* = 171 [66].

### 2.2. Constructs and Psychometric Instruments

All used measures were used in their adapted version to Brazilian and Portuguese contexts.

#### 2.2.1. Job Burnout

The BAT was used to assess burnout [29] through the development of two transculturally adapted versions: one for Brazil and one for Portugal (Table 1). The BAT-23 is a self-report psychometric instrument that comprises 23 items to be answered using a five-point rating scale (1—“Never”; 2—“Rarely”; 3—“Sometimes”; 4—“Often”; 5—“Always”). The BAT-23 version measures burnout’s core symptoms, and another version is also available that also includes items to assess burnout’s secondary symptoms. To develop the Portuguese version (Table 1), the BAT’s English version was used [34] following the ITC Guidelines for Translating and Adapting Tests [67]. BAT-23 operationalizes burnout as a second-order construct with four first-order factors: exhaustion (eight items), mental distance (five items), cognitive impairment (five items), and emotional impairment (five items). From BAT-23’s items, it is possible to extract a short version (BAT-12) with three items per each first-order latent construct (Table 1).

#### 2.2.2. Work Engagement

Work engagement refers to a positive motivational state and is composed of vigor, dedication, and absorption [68]. This construct was measured with the ultra-short version of the Utrecht Work Engagement Scale (UWES-3) [69], which used items from the short-version (i.e., UWES-9). The used UWES-3 items have been previously adapted with success to Portugal and Brazil [70,71]. It uses a seven-point ordinal scale (0—“Never”; 1—“Almost never”; 2—“Rarely”; 3—“Sometimes”; 4—“Often”; 5—“Very often”; 6—“Always”), with one item pertaining to each of the three dimensions. The UWES has shown good convergent evidence with the burnout scores since work engagement and burnout are moderately and negatively related [29,72]. The UWES already presented measurement invariance among Brazil and Portugal in the 9-item version [70]. One example of an item is: “At my work, I feel bursting with energy.”

#### 2.2.3. Co-Worker Support

Co-worker support refers to the function and quality of social relationships at work, such as perceived availability of help from coworkers or support actually received [73]. To assess the perceptions of co-worker support, the co-worker support sub-scale (3 items) of the Energy Compass psychometric instrument [74] was used. The items were answered using an ordinal five-point scale (1—“Never”; 2—“Seldom”; 3—“Sometimes”; 4—“Often”; 5—“Always”). One example of an item is: “Can you count on your colleagues for help and support when needed?”

#### 2.2.4. Role Clarity

The clarity of the role assesses the extent to which the tasks to be performed are clearly defined and the expectations and responsibilities for the employee are clear [75]. This construct was assessed using the sub-scale role clarity of the Energy Compass psychometric instrument [74]. The three items of the sub-scale were answered using a five-point ordinal scale (1—“Never”; 2—“Seldom”; 3—“Sometimes”; 4—“Often”; 5—“Always”). One example of an item is: “Is it sufficiently clear what you need to do in your job?”

#### 2.2.5. Work Overload

Work overload can be defined as the extent to which the employee has to deal with changes in job content, ICT systems, and leadership, as well as in the organization as a whole. Four items answered using a five-point ordinal scale (1—“Never”; 2—“Seldom”; 3—“Sometimes”; 4—“Often”; 5—“Always”) were used, as suggested in Schaufeli et al. [76]. One example of an item is, “Do you have too much work to do?”

#### 2.2.6. Negative Change

Negative change refers to the pessimistic views produced by the introduction of modifications at work, e.g., pace of work, interpersonal conflict, work-home conflict, and use of skills [77]. The negative change construct was assessed using the corresponding subscale (three items) from the Energy Compass psychometric instrument [74] answered in a five-point scale of frequency (1—“Never”; 2—“Seldom”; 3—“Sometimes”; 4—“Often”; 5—“Always”). An example of an item is, “Do changes cause turmoil in your company?”

### 2.3. Procedure

Data were collected simultaneously in Portugal and Brazil. The workers were invited to participate through social networks or e-mail. Firstly, the participants were presented with the electronic informed consent, which they had to accept to participate in the study. The digital survey was deployed using *LimeSurvey* [78] and *SurveyMonkey* [79], which contained a group of psychometric instruments together with a group of sociodemographic and job questions. To check how likely the research process would work, a pilot study was conducted with 15 workers, which provided feedback (e.g., potential issues with the digital platforms where the survey was deployed, clarity of the questions/items, and mean time of fulfillment).

All the subjects gave their informed consent for inclusion before they participated in the study. The study was conducted in accordance with the Declaration of Helsinki, and the study was approved by the Ethics Committee of the Federal University of Health Sciences of Porto Alegre Brazil, (CAAE 78617617.8.0000.5345; 25 October 2017).

### 2.4. Data Analysis

To conduct the statistical analysis the statistical programming language *R* [80] through the integrated development environment, *RStudio* [81] was used. To estimate the adequate sample size for the confirmatory factor analysis, the *MBESS* package [82] was used. The *skimr* package [83] and the *table1* package [84] were utilized to produce the descriptive statistics. The skewness (*sk*) using the “sample” method (i.e., sample skewness of the distribution) and the kurtosis (*ku*) using the “sample excess” method (i.e., sample kurtosis of the distribution with a value of 3 being subtracted) were calculated using the *PerformanceAnalytics* package [85]. The coefficient of variation (*CV*) was estimated with the *sjstats* package [86], the standard error of the mean (*SEM*) was calculated with the *plotrix* package [87]. The mode was computed by the *modeest* package [88]. Absolute values of |*sk*| > 3 and |*ku*| > 7 were considered as severe univariate normality violations [89,90]. To evaluate the multivariate normality the *psych* package [91] was used to calculate Mardia’s multivariate kurtosis [92].

To obtain evidence about the originally proposed dimensionality of the measurement models, the confirmatory factor analysis (CFA) was used. The following goodness-of-fit indices were used: *NFI* (Normed Fit Index), *TLI* (Tucker–Lewis Index), *CFI* (Comparative Fit Index), *RMSEA* (Root Mean Square Error of Approximation), and *SRMR* (Standardized Root Mean Square Residual). Estimates above 0.95 are considered good for *NFI*, *TLI*, and *CFI* [93]. While values below 0.08 were considered good for *SRMR* and *RMSEA* [94]. The package *lavaan* [95] was used to run the CFA analysis using the Weighted Least Squares Means and Variances (WLSMV) estimator [96]. The WLSMV was chosen because it does not require multivariate normality as an assumption, and because all items of the used psychometric instruments have an ordinal response scale.

The Average Variance Extracted (*AVE*) was estimated to test the evidence for convergent validity [97]. Satisfactory convergent validity evidence in terms of the internal structure was assumed for *AVE* ≥ 0.5 [98].

Item response theory analysis was conducted using a multidimensional polytomous Rasch model [99] as a particular case of the multidimensional random coefficients multinomial logit model (MRCMLM) [100]. The *TAM* package [101] was used to conduct the multidimensional polytomous Rasch analysis. Wright maps (also known as item-person maps or item maps) were used to present the location of both items and respondents on the same scale [102,103]. The *WrightMap* package [104] was used to produce the Wright Maps. Two mean square fit statistics (i.e., infit and outfit) were used to assess how well the data fit the model [105]. Considering the ordinal nature of the rating scale (i.e., 1—“Never” to 5—“Always”), the interval (0.6; 1.4) was considered as reasonable for the item mean square ranges for infit and outfit statistics [106]. Values above 1 suggest an increasing quantity of answers diverging from model’s predictions, while values below 1 indicate answers with less heterogeneity than expected [107].

To assess the evidence of reliability of the first-order factors, the following estimators of internal consistency were used: composite reliability (CR) [97], the α*_ordinal_* [47], and ω [108]. Values of ≥ 0.8 on the different mentioned estimators are considered indicative of acceptable reliability evidence [46,98]. The second-order latent factor also had estimates of internal consistency: the proportion of variance among first-order common factors that is attributable to the second-order factor (ω*_L_*_2_), the proportion of variance of a composite score calculated from the observed indicators that is attributable to the second-order factor (ω*_L_*_1_), and the proportion of observed variance explained by the second-order factor after partialling out the uniqueness from the first-order factors (ω*_partial L_*_1_). Both second-order and first-order internal consistency estimates were calculated using the *semTools* package [109]. In the item response theory framework, the MRCMLM provided the expected a posteriori (EAP) reliability index for each latent factor. The EAP reliability is defined as the ratio of the variance of the EAPs and the variance of the plausible values [110]. Values of EAP reliability ≥ 0.8 are preferable.

Using the theta-parameterization for categorical items through the *semTools* package [109], measurement invariance was evaluated comparing a group of eight different models [111]: (I) configural invariance, (II) thresholds of the indicators, (III) first-order factor loadings, (IV) structural weights, (V) intercepts of the first-order factors, (VI) latent means, (VII) disturbances of the first-order factors, and (VIII) residual variances of observed variables. The differences between the nested models were compared using two criteria. The Δ*CFI* ≤ −0.010 criterion [112], which advocates the non-rejection of the null hypothesis of invariance if the Δ*CFI* is smaller or equal to −0.010, and the Δχ^2^ criterion [113], which does not reject the null hypothesis of invariance if a significant χ^2^ robust difference test is obtained.

The structural models were tested using the *lavaan* package [95] to test validity evidence based on relations to other variables. In the latent score means comparison, Cohen’s *d* [114] was used as an effect size measure. The *doBy* package [115] was used to compute the raw score percentiles. A significance level of 5% was used (α = 0.05).

## 3. Results

### 3.1. Descriptive Statistics of Study Participants

A merged sample of 3103 workers was collected (*n_Brazil_* = 2217; *n_Portugal_* = 886) 74.2% female, with an average of 37.2 (11.1) years old. More than half of the workers (53.4%) were professionals according to the *International Standard Classification of Occupations* (ISCO-08) [116], and 72.5% had graduation or a higher academic level. Table 2 presents the descriptive statistics for each country, and for the merged sample.

### 3.2. Validity Evidence Base on the Internal Structure

This source of validity evidence investigates the dimensionality, reliability of the scores, and measurement invariance.

#### 3.2.1. Dimensionality

The distributional properties of BAT’s 23 items are presented in Table 3; these were used to judge distributional properties and psychometric sensitivity. None of the items in both countries presented severe univariate normality violations [89,90]. Mardia’s multivariate kurtosis [92] for the data from Brazil was 101.637 (*p* < 0.001), while for the data from Portugal it was 60.063 (*p* < 0.001). All items in both countries had the maximum range of possible answers, and no outliers were removed. These items’ distributional properties are indicative of appropriate psychometric sensitivity, as it would be expected that these items would follow an approximately normal distribution in the population under study. Despite these univariate normality indicators, the weighted least squares means and variances (WLSMV) [96] estimation method was used, taking into consideration the ordinal level of measurement of the items.

**Figure 1:**
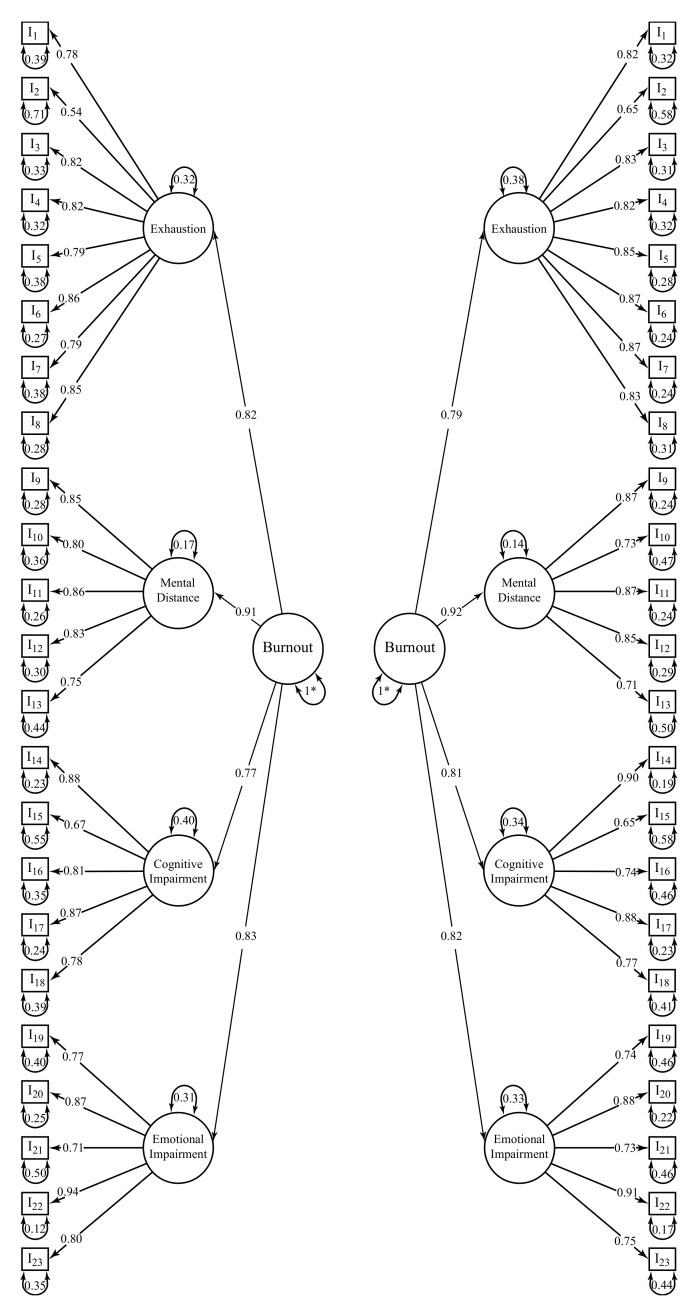
BAT-23’s factor loadings (λ) and structural weights (γ) for Brazil (**left**) and Portugal (**right**). * Indicates a fixed parameter. All paths were statistically significant (*p* < 0.001).

The BAT-23’s second-order latent factor model presented an acceptable fit (H1) to merged samples data (χ^2^_(226)_ = 4884.023; *p* < 0.001; *n* = 3103; *CFI* = 0.988; *NFI* = 0.988; *TLI* = 0.987; *SRMR* = 0.057; *RMSEA* = 0.082; *P*(RMSEA ≤ 0.05) < 0.001; 90% CI ]0.080; 0.084[). Both the fit the to the data from Brazil (Figure 1; χ^2^_(226)_ = 3398.828; *p* < 0.001; *n* = 2217; *CFI* = 0.988; *NFI* = 0.987; *TLI* = 0.987; *SRMR* = 0.058; *RMSEA* = 0.080; *P*(RMSEA ≤ 0.05) < 0.001; 90% CI ]0.077; 0.082[) and the fit to the data from Portugal (Figure 1; χ^2^_(226)_ = 1841.686; *p* < 0.001; *n* = 886; *CFI* = 0.987; *NFI* = 0.986; *TLI* = 0.986; *SRMR* = 0.064; *RMSEA* = 0.090; *P*(RMSEA ≤ 0.05) < 0.001; 90% CI ]0.086; 0.094[) was satisfactory. All factor loadings (*λ_i_* ≥ 0.57) and all structural weights (γ*_i_* ≥ 0.81) were satisfactory for the joint data.

**Figure 2:**
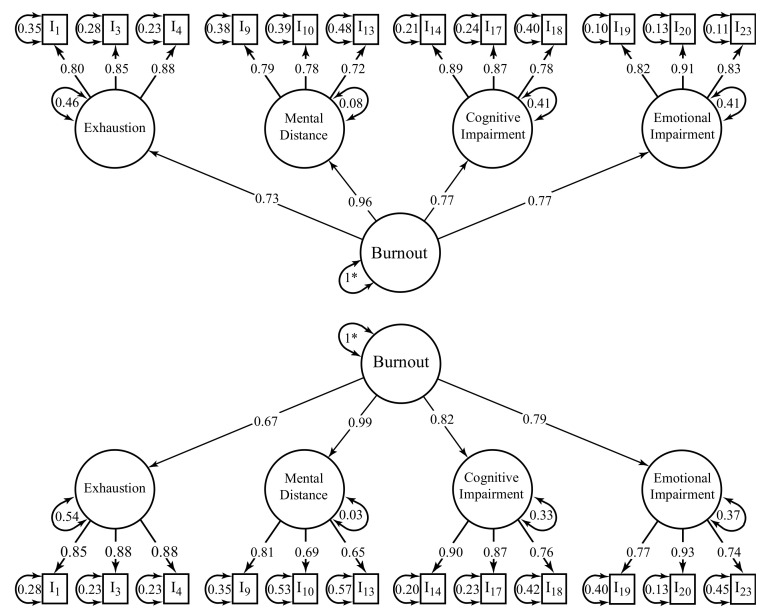
BAT-12’s factor loadings (λ) and structural weights (γ) for Brazil (**top**) and Portugal (**bottom**). * Indicates a fixed parameter. All paths were statistically significant (*p* < 0.001).

Regarding BAT-12, the fit to the data of the merged samples was satisfactory (H1; χ^2^_(50)_ = 521.809; *p* < 0.001; *n* = 3103; *CFI* = 0.996; *NFI* = 0.995; *TLI* = 0.994; *SRMR* = 0.037; *RMSEA* = 0.055; *P*(RMSEA ≤ 0.05) = 0.023; 90% CI ]0.051; 0.059[), as was the fit to the data of each country model, i.e., Brazil (Figure 2; χ^2^_(50)_ = 331.365; *p* < 0.001; *n* = 2217; *CFI* = 0.996; *NFI* = 0.995; *TLI* = 0.995; *SRMR* = 0.036; *RMSEA* = 0.050; *P*(RMSEA ≤ 0.05) = 0.439; 90% CI ]0.045; 0.056[) and Portugal (Figure 2; χ^2^_(50)_ = 300.631; *p* < 0.001; *n* = 886; *CFI* = 0.992; *NFI* = 0.991; *TLI* = 0.990; *SRMR* = 0.052; *RMSEA* = 0.075; *P*(RMSEA ≤ 0.05) < 0.001; 90% CI ]0.067; 0.084[).

All of BAT-23’s dimensions presented satisfactory *AVE* values both for the joint data (*AVE_Exhaustion_* = 0.63; *AVE_Mental Distance_* = 0.67; *AVE_Cognitive Impairment_* = 0.64; *AVE_Emotional Impairment_* = 0.67), and individually for Brazil (*AVE_Exhaustion_* = 0.62; *AVE_Mental Distance_* = 0.67; *AVE_Cognitive Impairment_* = 0.65; *AVE_Emotional Impairment_* = 0.68) and Portugal (*AVE_Exhaustion_* = 0.68; *AVE_Mental Distance_* = 0.65; *AVE_Cognitive Impairment_* = 0.63; *AVE_Emotional Impairment_* = 0.65). BAT-12 also showed satisfactory *AVE* estimates for the joint sample (*AVE_Exhaustion_* = 0.72; *AVE_Mental Distance_* = 0.57; *AVE_Cognitive Impairment_* = 0.72; *AVE_Emotional Impairment_* = 0.72), as it did for each individual countries’ sample: exhaustion (*AVE_BR_* = 0.71; *AVE_PT_* = 0.75), mental distance (*AVE_BR_* = 0.58; *AVE_PT_* = 0.52), cognitive impairment (*AVE_BR_* = 0.72; *AVE_PT_* = 0.72), and emotional impairment (*AVE_BR_* = 0.73; *AVE_PT_* = 0.67). The obtained *AVE* values are indicative of convergent evidence in terms of internal structure: on average the dimensions explain 62% or more (*AVE_i_* ≥ 0.62) of the variance of its indicators for BAT-23, and 52% or more of the variance of its indicators for BAT-12, i.e., *AVE_i_* ≥ 0.52 [98].

**Figure 3:**
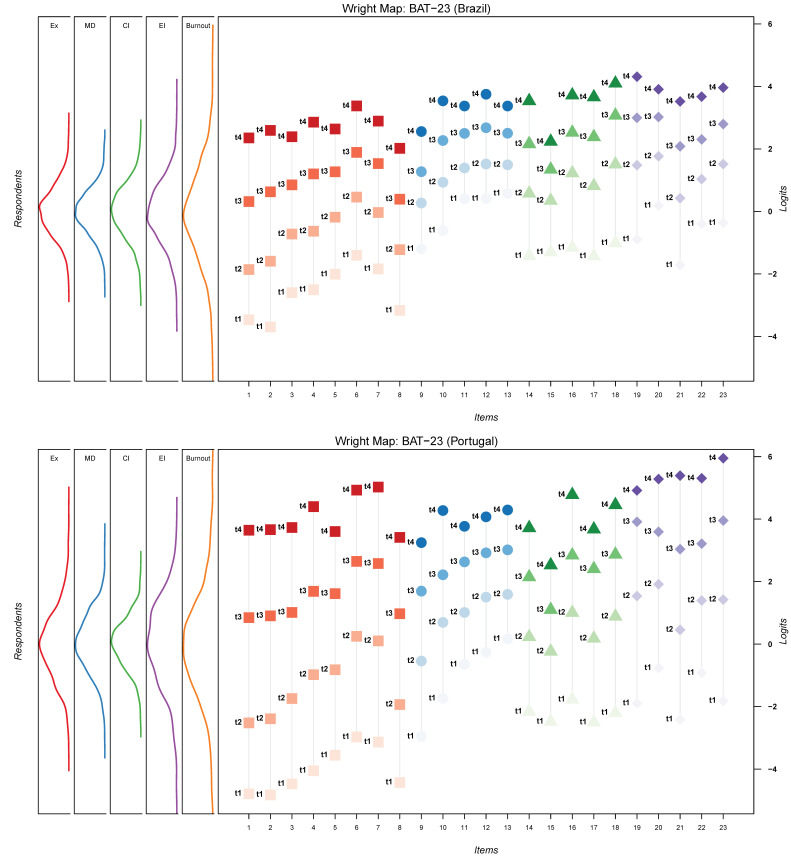
Wright Map for BAT-23 for both samples. Ex—Exhaustion; MD—Mental Distance; CI—Cognitive Impairment; CI—Emotional Impairment. Each item has its four thresholds identified (i.e., t1, t2, t3, t4).

From a Rasch perspective, the items match the workers’ sample, since BAT’s items garnered information about workers at all ranges of the burnout distribution. Figure 3 displays both items’ scale values (in terms of location) and persons’ burnout levels (in terms of their location) spaced along a common vertical axis marked with a logits scale [103,117].

#### 3.2.2. Reliability of the Scores: Internal Consistency

The reliability values of the second-order factor were good for Portugal (ω*_L_*_2_ = 0.90; ω*_L_*_1_ = 0.86; ω*_partial L_*_1_ = 0.97) and Brazil (ω*_L_*_2_ = 0.90; ω*_L_*_1_ = 0.87; ω*_partial L_*_1_ = 0.97), as they were for the joint sample (ω*_L_*_2_ = 0.90; ω*_L_*_1_ = 0.87; ω*_partial L_*_1_ = 0.97). BAT-12 presented similar values for Portugal (ω*_L_*_2_ = 0.96; ω*_L_*_1_ = 0.84; ω*_partial L_*_1_ = 0.94), Brazil (ω*_L_*_2_ = 0.92; ω*_L_*_1_ = 0.84; ω*_partial L_*_1_ = 0.94) and for the joint sample (ω*_L_*_2_ = 0.93; ω*_L_*_1_ = 0.84; ω*_partial L_*_1_ = 0.94). The internal consistency evidence of the second-order construct was very satisfactory when using the individual data from each country, as when using the joint data (H2). In the BAT-23 the *EAP* reliability estimate for the burnout latent score was 0.89 for Portugal, 0.74 for Brazil, and 0.88 for the joint sample. The BAT-12 model presented an *EAP* reliability estimate of 0.85 for Portugal, 0.84 for Brazil, and 0.85 for the joint sample. Regarding the internal consistency estimates of the first-order factors, all used estimators presented values, which were indicative of satisfactory reliability evidence for both BAT versions (H2; α*_ord i_* ≥ 0.76; ω*_i_* ≥ 0.71; *CR_i_* ≥ 0.76; Table 4). For both BAT-23 and BAT-12, the *EAP* reliability estimates for the four latent first-order factors were satisfactory (*EAP_i_* ≥ 0.82; Table 4).

#### 3.2.3. Measurement Invariance

The measurement invariance among countries and sex was tested through a group of nested models with increasing constraints (Table 5). Full uniqueness measurement invariance (i.e., strict invariance) was achieved among countries and sex for BAT-23 (H3) considering the Δ*CFI* ≤ −0.010 [112]. Using the Δχ^2^ criterion [113] thresholds, invariance among countries was achieved, and first-order factor loadings invariance was obtained among sex. However, the Δχ^2^ criterion is too restrictive [90]; consequently, the Δ*CFI* criterion was preferred. The fit of the data to the model was acceptable among countries and sex, as seen in the dimensionality analysis. The measurement of burnout using BAT works in a similar manner across countries and sex, allowing comparisons of scores to be established between the different groups.

BAT-12 presented scalar measurement invariance among workers from Brazil and Portugal (H3). In order to avoid negative disturbance (it is not theoretically possible) of the mental distance latent variable among the Portuguese sample, the disturbance of the mental distance first-order factor was constrained to 0.1 for three models (i.e., 4, 5, and, 6). Full uniqueness measurement invariance was achieved among sex using the Δ*CFI* criterion (H3).

The Pearson’s correlations between the raw BAT-12 and BAT-23 scores were very strong and statistically significant for the total burnout score (*r* = 0.979, *t*_(3,101)_ = 267.096; *p* < 0.001) and for all first-order dimensions, i.e., exhaustion (*r* = 0.932, *t*_(3,101)_ = 143.472; *p* < 0.001), mental distance (*r* = 0.960, *t*_(3,101)_ = 191.928; *p* < 0.001), cognitive impairment (*r* = 0.948, *t*_(3,101)_ = 165.722; *p* < 0.001), and emotional impairment (*r* = 0.948, *t*_(3,101)_ = 166.541; *p* < 0.001). The raw mean scores per dimension and for the total burnout score are presented (Table 6 and Table 7).

### 3.3. Validity Evidence Based on the Relations to Other Variables

The latent correlations among BAT’s burnout latent factor with other related latent variables were investigated. Regarding the sample from Brazil, the measurement model with burnout-related constructs provided a satisfactory fit to the data when using either BAT-23 (χ^2^_(683)_ = 5501.555; *p* < 0.001; *n* = 1006; *CFI* = 0.978; *NFI* = 0.975; *TLI* = 0.976; *SRMR* = 0.074; *RMSEA* = 0.084; *P*(RMSEA ≤ 0.05) < 0.001; 90% CI ]0.082; 0.086[) or BAT-12 (χ^2^_(331)_ = 2454.727; *p* < 0.001; *n* = 1006; *CFI* = 0.978; *NFI* = 0.975; *TLI* = 0.976; *SRMR* = 0.073; *RMSEA* = 0.080; *P*(RMSEA ≤ 0.05) < 0.001; 90% CI ]0.077; 0.083[). The data of the workers’ sample from Portugal also presented a satisfactory fit to the data when using either BAT-12 (χ^2^_(331)_ = 1150.641; *p* < 0.001; *n* = 355; *CFI* = 0.987; *NFI* = 0.982; *TLI* = 0.985; *SRMR* = 0.076; *RMSEA* = 0.084; *P*(RMSEA ≤ 0.05) < 0.001; 90% CI ]0.078; 0.089[) or BAT-23 (χ^2^_(683)_ = 2691.560; *p* < 0.001; *n* = 355; *CFI* = 0.988; *NFI* = 0.983; *TLI* = 0.986; *SRMR* = 0.076; *RMSEA* = 0.091; *P*(RMSEA ≤ 0.05) < 0.001; 90% CI ]0.088; 0.095[). In terms of reliability, the internal consistency estimates obtained ranged from acceptable to good: work engagement (ω*_Brazil_* = 0.86; ω*_Portugal_* = 0.74), co-worker support (ω*_Brazil_* = 0.84; ω*_Portugal_* = 0.86), role clarity (ω*_Brazil_* = 0.83; ω*_Portugal_* = 0.89), negative change (ω*_Brazil_* = 0.68; ω*_Portugal_* = 0.79), and work overload (ω*_Brazil_* = 0.85; ω*_Portugal_* = 0.84).

The models’ latent correlations were virtually the same whether using BAT-23 or BAT-12 within each country (Table 8). The sample from Brazil presented BAT’s latent burnout score correlations ranging from −0.81 (work engagement) to 0.59 (work overload) when using BAT-23, and they ranged from −0.80 (work engagement) to 0.61 (work overload) when using BAT-12. For the Portuguese sample, the latent burnout scores’ correlations ranged from −0.75 with work engagement to 0.54 with negative change for BAT-23. For the same sample, BAT-12’s burnout latent correlations varied from −0.73 (work engagement) to 0.54 (negative change). All latent correlations with BAT’s burnout score were statistically significant (Table 8) and presented a moderate to strong effect size (H4) [114].

## 4. Discussion

The data from Brazil and Portugal provided robust validity evidence for the BAT-23, and the BAT-12 using item response theory (i.e., multidimensional Rasch model) and classical test theory (i.e., confirmatory factor analysis) in conjunction. Satisfactory evidence was obtained based on both the internal structure and the relations to other variables. The current study adds to the already available evidence about BAT’s psychometric properties using the classical test theory, e.g., [21], and item response theory [37]. The present study intends to take advantage of the benefits of the two measurement theories in conjunction while bringing some novelties, such as the second-order estimates of internal consistency (i.e., ω*_L_*_2_, ω*_L_*_1_, and ω*_partial L_*_1_) and the EAP reliability index. In terms of the Rasch model, the MRCMLM was used in contrast with the unidimensional approach used in BAT’s previous research [37]. The multidimensional measurement model is both substantively advantageous and technically appropriate in cases where the unidimensionality is not expected [99]. The multidimensional approach considered BAT’s four first-order dimensions, and a second-order latent variable. This is also the first study to provide infit and outfit estimates for BAT’s items, these two mean square statistics are useful to understand how well the data fit the model [107].

The originally proposed dimensionality for BAT-23 and BAT-12 presented a satisfactory fit to the data for both countries without removing items (H1). Such findings are corroborated by samples from other American and European countries, Ecuador using BAT-23 and BAT-12 [40], and Italy using BAT-23 [38]. Currently, the cumulated evidence of BAT’s dimensionality is consistent across countries from Asia, America, and Europe [21].

Globally, the evidence of the reliability of the scores in terms of internal consistency obtained by both samples was satisfactory both for second- and first-order dimensions (H2). In fact, only the mental distance dimension of the BAT-12 version with the Portuguese data presented estimates slightly below the desirable the Portuguese workers; nevertheless, those values were acceptable (i.e., ≥0.71). BAT’s mental distance was the first-order dimension that had the lowest α, and ω in the Ecuadorian version [40], as did in the Italian version [38]. However, samples from other countries showed that mental distance did not present the lowest internal consistency estimates of all first-order dimensions [21]. As expected, BAT-12’s first-order internal consistency estimates were lower than BAT-23’s ones. Notwithstanding, BAT-12’s internal consistency estimates were globally satisfactory [46], with both classical test theory (i.e., α*_ord_*, ω, and *CR*) and item theory response estimators (i.e., *EAP*).

Both versions of BAT had measurement invariance (i.e., at least scalar) for countries and sex (H3), allowing mean comparisons for BAT among countries, and sex. BAT-23 presented measurement invariance among seven countries in a previous study by De beer et al. [21]. One of the novelties of the current study is the measurement invariance of BAT-23 among sex and the test of measurement invariance among countries and sex of BAT’s short version (i.e., BAT-12).

The BAT’s scores’ relation to other variables presented convergent evidence (H4) since all latent correlations’ paths were statistically significant (moderate to strong effect sizes) with the theoretically expected direction for each correlation pair. BAT’s burnout latent scores were negatively correlated with work engagement, role clarity, and co-workers’ support. Positive latent correlations were found among BAT’s burnout latent scores, work overload, and negative change. The latent correlations’ effect sizes were similar among countries. Burnout’s correlation with work overload and burnout’s correlation with role clarity presented the largest difference among countries. The observed latent associations between BAT’s scores and job demands and job resources are in accordance with the findings from research reading other BAT’s versions; for example, the Romanian [39] and the Japanese [28]. BAT’s burnout latent scores’ correlation with work engagement was the strongest negative correlation for both countries. Strong negative correlations between burnout and work engagement are in accordance with what is theoretically expected from these two constructs [58,118]. Regarding the data from Brazil, the strongest positive correlation with BAT’s burnout scores was achieved with work overload. While for Portugal, the strongest correlation for BAT’s burnout latent scores was observed with negative change. However, the absolutes values were smaller than the ones observed for burnout and work engagement correlation. The data provided validity evidence based on the relations to other variables, allowing to consistently build up on the existing burnout’s nomological network of constructs [56] reinforcing BAT’s psychometric properties.

Both BAT-23 and BAT-12 presented good validity evidence and thus both can be used to measure burnout levels among workers from Brazil and from Portugal. The advantage of using BAT-23 concerns its finer-grained assessment of burnout (i.e., more items lead to capturing more content of the construct); however, if time is a constraint and other measures are being collected, BAT-12 can be a more parsimonious alternative. As the results showed, the obtained raw scores for BAT-12 and BAT-23 presented an almost perfect correlation (i.e., 0.98). Moreover, short-versions of psychometric instruments are preferable since their validity evidence is not compromised with its shorting from the full-length version. The main goal of using a short-version is to reduce the time burden of assessment. Usually, short-versions have lower estimates of reliability than full versions. The BAT-12 results were (in some of the first-order dimensions) slightly lower than the BAT-23 ones, although with no meaningful losses in terms of its satisfactory validity evidence. Practitioners and researchers opting between BAT-23 and BAT-12 will have to balance between time-saving of brevity versus construct content coverage and validity evidence [119]. The BAT-12 option seems to be the most balanced, since its validity evidence is equivalent to its longer counterpart, and longer instruments can present several problems, for example, boredom, fatigue, increasing dropout rates, and lack of attention [120].

### 4.1. Weaknesses, Strengths, and Suggestions for Further Research

The obtained non-probabilistic convenience sample introduces some degree of selection bias. However, probabilistic sampling (i.e., all units in the population have known and positive probabilities of inclusion) is only possible when there is a complete and up-to-date list of the member of the population being investigated [121,122], which was not the case. Even with large samples, the representativeness of the samples cannot be assumed if the sampling method is not probabilistic. However, many valid conclusions can still be taken from the current study. Future research should be conducted with samples from occupational groups with few elements in the current paper (e.g., craft and related trades workers or elementary occupations). The sex proportions and academic level should also be more similar to each workers’ population parameters. The current correlational study has a cross-sectional design. Longitudinal designs can strengthen the validity evidence of BAT, namely allowing longitudinal measurement invariance to be tested, which will allow BAT’s structure stability through time to be studied. The current paper only investigated two of the five sources of validity evidence [43]. One of them (i.e., validity evidence based on the relations to other variables) was only analyzed from a correlational perspective with five related constructs. Further research on the BAT’s scores’ relations to other variables should expand to other conceptually linked constructs such as fatigue using, for example, the Portuguese adaptation of the Occupational Fatigue Exhaustion/Recovery [6] or the Brazilian adaptation of the Feeling of Fatigue scale [123]. Test-criterion relationships should be analyzed in future studies using a predictive or concurrent design. Future studies should also investigate other sources of validity evidence, e.g., the validity evidence based on the response processes.

The findings of the current study are based on large samples from two different countries. The obtained findings are promising in terms of the measurement of burnout’s core symptoms. Future research should investigate the version of BAT including the secondary symptoms items for both countries, so as to also compare BAT’s psychometric properties directly with the Brazilian and Portuguese adaptations of other psychometric instruments that measure burnout (e.g., MBI, OLBI, CBI). It will also be convenient to obtain cut-off values for different levels of burnout; for such a purpose, clinical samples will have to be investigated. Using the receiver operating characteristic (ROC) curve [124] will allow sensitivity (true positive rate) and specificity (true negative rate) to be estimated. Although it should be taken into consideration that BAT’s score by themselves will not be enough, a full thorough clinical interview and complementary information will be required [29]. Another call that should be made for future studies is the incorporation of the increasing evidence (prior knowledge) about BAT’s dimensionality to take advantage of the Bayesian approach [125], which particularly useful with small samples and allows some frequentist approach potential problems to be avoided (e.g., non-convergence, negative variances).

### 4.2. Practical Implications

BAT-12 was shown to be virtually equal to BAT-23 in terms of scores, as in terms of validity evidence, representing an equally robust alternative to measure burnout. The decision to use BAT-23 or BAT-12 will be related to the level of detail that one intends to obtain regarding the burnout measurement. BAT’s Brazilian and Portuguese versions are invariant in terms of measurement, allowing for comparisons of means among countries, and between males and females. BAT’s scores presented the expected associations with related measures. The quartiles and mean scores are also provided as the first reference in terms of burnout at the country and sex levels. BAT is a promising instrument and is a viable alternative to measuring burnout in workers from Brazil and Portugal.

## 5. Conclusions

The data of multi-occupational workers from Brazil and Portugal presented good validity evidence for both BAT-23 and BAT-12, supporting its use to measure and to compare burnout levels among sex and countries. BAT’s scores provided support for the theoretical nomological network of constructs. Both samples’ data fitted well the original structure of BAT-23 and BAT-12 with good reliability evidence. This leads to the conclusion that BAT is a good instrument for practitioners and researchers to measure burnout among different occupations.

## Figures and Tables

**Table 1:** BAT English, Brazilian, and Portuguese adaptations.

Item	English					Brazil			Portugal	
	Never	Rarely	Sometimes	Often	Always	Nunca	Raramente	Algumas vezes	Frequentemente	Sempre
	1	2	3	4	5	1	2	3	4	5
Exhaustion						Exaustão				
1 *^S^*	At work, I feel mentally exhausted					No trabalho, sinto-me mentalmente exausto			No trabalho, sinto-me mentalmente exausto(a)	
2	Everything I do at work requires a great deal of effort					Tudo o que faço no trabalho exige muito esforço			Tudo o que faço no trabalho exige muito esforço	
3 *^S^*	After a day at work, I find it hard to recover my energy					Acho difícil recuperar minha energia depois de um dia de trabalho			Depois de um dia no trabalho, acho difícil recuperar a minha energia	
4 *^S^*	At work, I feel physically exhausted					No trabalho, sinto-me fisicamente exausto			No trabalho, sinto-me fisicamente exausto(a)	
5	When I get up in the morning, I lack the energy to start a new day at work					Ao levantar pela manhã, me falta energia para começar um novo dia no trabalho			Quando me levanto de manhã, falta-me a energia para começar um novo dia no trabalho	
6	I want to be active at work, but somehow I am unable to manage					Quero ser ativo no trabalho, mas de alguma forma não consigo			Quero estar ativo(a) no trabalho, mas de alguma forma sou incapaz de o fazer	
7	When I exert myself at work, I get tired quicker than normal					Quando eu me esforço no trabalho, me canso mais rápido do que o normal			Quando me esforço no trabalho, fico rapidamente cansado(a)	
8	At the end of my working day, I feel mentally exhausted and drained					No final do meu dia de trabalho, eu me sinto mentalmente exausto e esgotado			No final de um dia de trabalho, sinto-me mentalmente exausto(a) e esgotado(a)	
Mental distance						Distância mental				
9 *^S^*	I struggle to find any enthusiasm for my work					Eu luto para encontrar algum entusiasmo pelo meu trabalho			Tenho dificuldade em encontrar algum entusiasmo pelo meu trabalho	
10 *^S^*	At work, I do not think what I am doing and I function on autopilot					Não penso no que estou fazendo no meu trabalho, eu funciono em piloto automático			No trabalho, não penso muito no que estou a fazer e funciono em piloto automático	
11	I feel a strong aversion towards my job					Sinto forte aversão pelo meu trabalho			Sinto uma forte aversão em relação ao meu trabalho	
12	I feel indifferent about my job					Sinto-me indiferente em relação ao meu trabalho			Sinto-me indiferente em relação ao meu trabalho	
13 *^S^*	I am cynical about what my work means to others					Sou pessimista sobre o que meu trabalho significa para os outros			Sou cínico(a) sobre o que o meu trabalho significa para os outros	
Cognitive impairment						Incapacidade no Controlo Cognitivo				
14 *^S^*	At work, I have trouble staying focused					Em meu trabalho, tenho dificuldade em manter o foco			No trabalho, tenho dificuldade em manter-me focado(a)	
15	At work I struggle to think clearly					No trabalho, eu me esforço para pensar claramente			No trabalho, luto para pensar claramente	
16	I am forgetful and distracted at work					Sou esquecido e distraído no trabalho			Sou esquecido(a) e distraído(a) no trabalho	
17 *^S^*	When I’m working, I have trouble concentrating					Tenho dificuldade em me concentrar quando estou trabalhando			Quando estou a trabalhar, tenho dificuldade em me concentrar	
18 *^S^*	I make mistakes in my work because I have my mind on other things					Cometo erros no trabalho porque minha mente está em outras coisas			Faço erros no meu trabalho porque tenho a cabeça sobrecarregada com outras coisas	
Emotional impairment						Incapacidade no Controlo Emocional				
19 *^S^*	At work, I feel unable to control my emotions					No trabalho, sinto-me incapaz de controlar as minhas emoções			No trabalho, sinto-me incapaz de controlar as minhas emoções	
20 *^S^*	I do not recognize myself in the way I react emotionally at work					Eu não me reconheço na maneira como reajo emocionalmente no trabalho			Não me reconheço na maneira como reajo emocionalmente no trabalho	
21	During my work I become irritable when things do not go the way I want					Durante o trabalho, fico irritado quando as coisas não são do jeito que eu quero			Durante o trabalho, fico irritadiço(a) quando as coisas não são como eu quero	
22	I get upset and sad at work without knowing why					Fico insatisfeito e triste no trabalho sem saber o porquê			Fico perturbado(a) e triste no trabalho sem saber porquê	
23 *^S^*	At work I may overreact unintentionally					No trabalho, eu posso ter reações exageradas sem querer			Pode acontecer que no trabalho eu reaja exageradamente sem querer	

Note. *^S^*—short version (BAT-12).

**Table 2:** Descriptive statistics of the samples’ sociodemographic, and occupational group variables.

	Brazil	Portugal	Total
	(*n* = 2217)	(*n* = 886)	(*N* = 3103)
**Age (Years)**			
*M* (*SD*)	36.9 (11.1)	38.9 (11.4)	37.2 (11.1)
*Mdn* [*Min*, *Max*]	36.0 [17.0, 90.0]	41.0 [18.0, 68.0]	36.0 [17.0, 90.0]
**Sex**			
Female	1653 (74.8%)	537 (72.5%)	2190 (74.2%)
Male	558 (25.2%)	204 (27.5%)	762 (25.8%)
**Academic Level**			
High school, vocational education, or lower	554 (25.0%)	258 (34.6%)	812 (27.4%)
Graduation	547 (24.7%)	204 (27.3%)	751 (25.3%)
Post-graduation	1116 (50.3%)	284 (38.1%)	1400 (47.2%)
**Occupational Group (ISCO-08)**			
Armed Forces Occupations	6 (0.3%)	0 (0.0%)	6 (0.2%)
Clerical Support Workers	1 (0.1%)	133 (20.2%)	134 (5.2%)
Craft and Related Trades Workers	1 (0.1%)	5 (0.8%)	6 (0.2%)
Elementary Occupations	15 (0.8%)	4 (0.6%)	19 (0.7%)
Managers	149 (7.8%)	63 (9.6%)	212 (8.3%)
Professionals	1071 (56.2%)	300 (45.5%)	1371 (53.4%)
Services and Sales Workers	75 (3.9%)	83 (12.6%)	158 (6.2%)
Technicians and Associate Professionals	589 (30.9%)	65 (9.9%)	654 (25.5%)
Plant and Machine Operators and Assemblers	0 (0.0%)	7 (1.1%)	7 (0.3%)

Note. Because of rounding, some results may appear inconsistent.

**Table 3:** BAT items’ infit, outfit statistics, and distributional properties for Portugal and Brazil.

Item	*M*	*SD*	*Min*	*P*_25_	*Mdn*	*P*_75_	*Max*	Histogram	*SEM*	*CV*	*Mode*	*sk*	*ku*	*Infit*	*Outfit*
**Brazil**															
Item 1 *^S^*	3.31	0.99	1	3	3	4	5	▁▃▇▆▂	0.02	0.30	3	−0.23	−0.22	0.981	0.977
Item 2	3.19	0.97	1	3	3	4	5	▁▃▇▅▂	0.02	0.30	3	−0.05	−0.31	1.412	1.434
Item 3 *^S^*	2.94	1.12	1	2	3	4	5	▂▆▇▅▂	0.02	0.38	3	0.09	−0.71	0.924	0.920
Item 4 *^S^*	2.82	1.06	1	2	3	3	5	▂▆▇▅▂	0.02	0.38	3	0.13	−0.51	0.897	0.884
Item 5	2.67	1.14	1	2	3	3	5	▅▇▇▅▂	0.02	0.43	2	0.32	−0.63	1.075	1.087
Item 6	2.32	1.06	1	2	2	3	5	▆▇▆▂▁	0.02	0.46	2	0.56	−0.30	1.099	1.109
Item 7	2.56	1.10	1	2	2	3	5	▅▇▇▃▂	0.02	0.43	2	0.37	−0.50	1.043	1.033
Item 8	3.20	1.11	1	2	3	4	5	▂▅▇▆▃	0.02	0.35	3	−0.09	−0.71	0.871	0.857
Item 9 *^S^*	2.47	1.27	1	1	2	3	5	▇▇▆▅▂	0.03	0.51	2	0.49	−0.84	1.115	1.108
Item 10 *^S^*	2.05	1.07	1	1	2	3	5	▇▆▅▂▁	0.02	0.52	1	0.84	−0.01	1.139	1.132
Item 11	1.75	1.05	1	1	1	2	5	▇▃▂▁▁	0.02	0.60	1	1.38	1.14	0.975	1.028
Item 12	1.71	1.00	1	1	1	2	5	▇▃▂▁▁	0.02	0.58	1	1.39	1.28	0.994	0.935
Item 13 *^S^*	1.69	1.04	1	1	1	2	5	▇▂▂▁▁	0.02	0.61	1	1.50	1.50	1.285	1.422
Item 14 *^S^*	2.27	1.01	1	2	2	3	5	▅▇▆▂▁	0.02	0.44	2	0.56	−0.15	0.919	0.902
Item 15	2.47	1.24	1	1	2	3	5	▆▇▆▃▂	0.03	0.50	2	0.58	−0.63	1.562	1.685
Item 16	2.05	0.93	1	1	2	3	5	▅▇▃▁▁	0.02	0.45	2	0.87	0.66	0.985	0.983
Item 17 *^S^*	2.19	0.96	1	2	2	3	5	▅▇▅▁▁	0.02	0.44	2	0.66	0.18	0.885	0.869
Item 18 *^S^*	1.95	0.85	1	1	2	2	5	▆▇▃▁▁	0.02	0.43	2	0.86	0.92	0.994	0.981
Item 19 *^S^*	1.95	0.91	1	1	2	2	5	▇▇▃▁▁	0.02	0.47	2	0.92	0.71	1.061	1.075
Item 20 *^S^*	1.71	0.93	1	1	1	2	5	▇▅▂▁▁	0.02	0.55	1	1.40	1.69	0.956	0.889
Item 21	2.35	1.04	1	2	2	3	5	▅▇▆▂▁	0.02	0.44	2	0.54	−0.23	1.185	1.190
Item 22	1.99	1.08	1	1	2	3	5	▇▆▃▂▁	0.02	0.54	1	0.93	0.06	1.047	1.019
Item 23 *^S^*	1.86	0.96	1	1	2	2	5	▇▆▃▁▁	0.02	0.52	1	1.12	0.89	0.975	0.980
**Portugal**															
Item 1 *^S^*	3.21	0.86	1	3	3	4	5	▁▂▇▅▁	0.03	0.27	3	−0.09	0.22	0.927	0.930
Item 2	3.18	0.86	1	3	3	4	5	▁▂▇▅▁	0.03	0.27	3	−0.04	0.12	1.313	1.312
Item 3 *^S^*	3.07	0.92	1	2	3	4	5	▁▅▇▅▁	0.03	0.30	3	0.03	−0.24	0.900	0.907
Item 4 *^S^*	2.84	0.91	1	2	3	3	5	▁▆▇▃▁	0.03	0.32	3	0.17	−0.22	0.912	0.905
Item 5	2.82	1.00	1	2	3	3	5	▂▆▇▃▁	0.03	0.36	3	0.26	−0.29	0.979	0.979
Item 6	2.45	0.91	1	2	2	3	5	▂▇▆▂▁	0.03	0.37	2	0.46	0.04	0.988	0.989
Item 7	2.49	0.90	1	2	2	3	5	▂▇▆▂▁	0.03	0.36	2	0.41	−0.02	0.888	0.879
Item 8	3.12	0.93	1	3	3	4	5	▁▃▇▅▁	0.03	0.30	3	0.02	−0.16	0.913	0.903
Item 9 *^S^*	2.75	0.99	1	2	3	3	5	▂▆▇▃▁	0.03	0.36	3	0.24	−0.25	0.979	0.986
Item 10 *^S^*	2.29	0.99	1	2	2	3	5	▅▇▅▂▁	0.03	0.43	2	0.54	−0.24	1.152	1.148
Item 11	2.02	1.03	1	1	2	3	5	▇▇▅▁▁	0.03	0.51	1	0.84	0.15	0.859	0.878
Item 12	1.85	0.96	1	1	2	2	5	▇▆▃▁▁	0.03	0.52	1	1.08	0.72	0.876	0.772
Item 13 *^S^*	1.75	0.95	1	1	1	2	5	▇▅▂▁▁	0.03	0.55	1	1.21	0.91	1.257	1.339
Item 14 *^S^*	2.42	0.94	1	2	2	3	5	▃▇▆▂▁	0.03	0.39	2	0.40	−0.06	0.816	0.808
Item 15	2.76	1.12	1	2	3	4	5	▃▇▇▅▂	0.04	0.41	2	0.30	−0.67	1.310	1.349
Item 16	2.16	0.84	1	2	2	3	5	▃▇▃▁▁	0.03	0.39	2	0.56	0.29	0.970	0.961
Item 17 *^S^*	2.45	0.88	1	2	2	3	5	▂▇▆▂▁	0.03	0.36	2	0.43	0.29	0.771	0.776
Item 18 *^S^*	2.23	0.81	1	2	2	3	5	▂▇▅▁▁	0.03	0.36	2	0.56	0.53	0.960	0.944
Item 19 *^S^*	2.08	0.81	1	2	2	3	5	▃▇▃▁▁	0.03	0.39	2	0.72	1.01	1.150	1.161
Item 20 *^S^*	1.87	0.87	1	1	2	2	5	▇▇▃▁▁	0.03	0.47	2	0.96	0.82	0.855	0.821
Item 21	2.34	0.89	1	2	2	3	5	▃▇▆▂▁	0.03	0.38	2	0.33	−0.17	1.076	1.085
Item 22	1.99	0.94	1	1	2	3	5	▇▇▅▁▁	0.03	0.47	2	0.77	0.08	0.981	0.929
Item 23 *^S^*	2.07	0.81	1	2	2	3	5	▃▇▃▁▁	0.03	0.39	2	0.54	0.25	0.979	0.987

Note. *^S^*—short version (BAT-12).

**Table 4:** Reliability Estimates for the Sample from Brazil, Portugal, and Joint Data.

BAT-23												
BAT Dimensions	Brazil				Portugal				Total			
	α*_ord_*	ω	*CR*	*EAP*	α*_ord_*	ω	*CR*	*EAP*	α*_ord_*	ω	*CR*	*EAP*
Exhaustion	0.92	0.91	0.95	0.92	0.92	0.91	0.96	0.92	0.93	0.92	0.96	0.92
Mental Distance	0.91	0.88	0.93	0.88	0.91	0.88	0.93	0.89	0.90	0.88	0.93	0.88
Cognitive Impairment	0.89	0.86	0.92	0.86	0.89	0.86	0.91	0.87	0.89	0.86	0.92	0.87
Emotional Impairment	0.91	0.88	0.95	0.87	0.91	0.88	0.94	0.88	0.90	0.88	0.94	0.88
**BAT-12**												
**BAT Dimensions**	**Brazil**				**Portugal**				**Total**			
	**α** *_ord_*	**ω**	***CR***	***EAP***	**α** *_ord_*	**ω**	***CR***	***EAP***	**α** *_ord_*	**ω**	***CR***	***EAP***
Exhaustion	0.88	0.85	0.88	0.85	0.90	0.86	0.90	0.85	0.88	0.85	0.88	0.85
Mental Distance	0.81	0.76	0.81	0.84	0.76	0.71	0.76	0.83	0.80	0.75	0.80	0.84
Cognitive Impairment	0.87	0.85	0.88	0.83	0.86	0.84	0.88	0.84	0.87	0.85	0.88	0.84
Emotional Impairment	0.89	0.85	0.89	0.82	0.86	0.81	0.86	0.82	0.88	0.84	0.88	0.82

**Table 5:** Measurement Invariance (for BAT-23 and BAT-12) Between Countries, and Sex.

BAT-23					
Countries (*n_Brazil_* = 2217, and *n_P__ortugal_* = 886)					
Model Invariance	χ^2^*_scaled_*	*df*	*CFI_scaled_*	Δχ^2^	Δ*CFI_scaled_*
1—Configural	6635.928	452	0.934	-	-
2—Thresholds	6725.056	494	0.934	50.517 *^ns^*	−0.000
3—Factor loadings	6677.411	513	0.934	76.445 ***	0.000
4—Structural weights	6303.609	516	0.938	8.782 *	0.004
5—Intercepts (first-order)	6558.480	539	0.936	239.490 ***	−0.002
6—Latent means	6572.809	543	0.936	94.758 ***	0.000
7—Disturbances	7064.532	547	0.930	15.470 **	−0.006
8—Residuals	6371.645	570	0.938	204.445 ***	−0.008
**Sex (*n_Female_* = 2190, and *n_Male_* = 762)**					
1—Configural	6325.536	452	0.934	-	-
2—Thresholds	6458.035	494	0.933	53.446 *^ns^*	−0.001
3—Factor loadings	6319.252	513	0.935	21.808 *^ns^*	0.002
4—Structural weights	6034.940	516	0.938	9.471 *	0.003
5—Intercepts (first-order)	6076.032	539	0.938	78.902 ***	0.000
6—Latent means	5163.396	543	0.948	16.141 **	0.010
7—Disturbances	5973.735	547	0.939	59.214 ***	−0.009
8—Residuals	5407.522	570	0.946	147.978 ***	0.007
**BAT-12**					
**Countries (*n_Brazil_* = 2217, and *n_P_*** ***_ortugal_* = 886)**					
**Model Invariance**	**χ^2^*_scaled_***	***df***	***CFI_scaled_***	**Δχ^2^**	**Δ*CFI_scaled_***
1—Configural	1043.678	100	0.977	-	-
2—Thresholds	1080.661	120	0.977	26.138 *^ns^*	0.000
3—Factor loadings	1081.793	128	0.977	21.379 **	0.000
4—Structural weights	1032.024	132	0.978	16.411 **	0.001
5—Intercepts (first-order)	1170.409	144	0.975	113.210 ***	−0.003
6—Latent means	1750.003	148	0.961	127.530 ***	−0.014
7—Disturbances	1690.957	151	0.963	-132.455 *^ns^*	0.002
8—Residuals	1763.487	163	0.961	118.999 ***	−0.002
**Sex (*n_Female_* = 2190, and *n_Male_* = 762)**					
1—Configural	930.282	100	0.979	-	-
2—Thresholds	970.210	120	0.979	23.217 *^ns^*	0.000
3—Factor loadings	958.424	128	0.979	9.269 *^ns^*	0.000
4—Structural weights	911.328	131	0.980	9.925 *	0.001
5—Intercepts (first-order)	973.760	143	0.979	44.065 ***	−0.001
6—Latent means	845.688	147	0.982	15.721 **	0.003
7—Disturbances	1172.354	151	0.974	46.429 ***	−0.008
8—Residuals	1173.808	163	0.975	57.478 ***	0.001

Note. ^*ns*^ *p* > 0.05; * *p* ≤ 0.05; ** *p* ≤ 0.01; *** *p* < 0.001. The χ^2^*_scaled_* column contains the robust test. The Δχ^2^ column contains the robust difference test which is a function of two standard (not robust) statistics.

**Table 6:** BAT’s raw mean scores distributional properties (workers from Brazil).

BAT−23										
Brazil Total Sample (*n* = 2217)										
Dimension	*M*	*SD*	*Min*	*P*_25_	*Mdn*	*P*_75_	*Max*	Histogram	*sk*	*ku*
Exhaustion	2.87	0.83	1.00	2.25	2.88	3.38	5.00	▂▅▇▃▂	0.18	−0.27
Mental distance	1.93	0.87	1.00	1.20	1.80	2.40	5.00	▇▃▂▁▁	1.09	0.78
Cognitive impairment	2.19	0.79	1.00	1.60	2.20	2.60	5.00	▇▇▃▁▁	0.62	0.43
Emotional impairment	1.97	0.81	1.00	1.40	1.80	2.40	5.00	▇▅▂▁▁	1.08	1.17
Burnout	2.32	0.69	1.00	1.83	2.26	2.74	5.00	▅▇▅▁▁	0.68	0.58
**Brazil Female Sample (*n* = 1653)**										
Exhaustion	2.90	0.83	1.00	2.38	2.88	3.38	5.00	▂▅▇▃▂	0.15	−0.30
Mental distance	1.90	0.86	1.00	1.20	1.60	2.40	5.00	▇▃▂▁▁	1.11	0.79
Cognitive impairment	2.19	0.78	1.00	1.60	2.00	2.60	5.00	▇▇▃▁▁	0.59	0.31
Emotional impairment	1.99	0.80	1.00	1.40	1.80	2.40	5.00	▇▅▂▁▁	1.01	0.95
Burnout	2.33	0.69	1.00	1.83	2.26	2.74	5.00	▅▇▅▁▁	0.65	0.37
**Brazil Male Sample (*n* = 558)**										
Exhaustion	2.78	0.83	1.00	2.25	2.75	3.38	5.00	▂▆▇▃▁	0.26	−0.13
Mental distance	2.02	0.90	1.00	1.40	1.80	2.60	5.00	▇▃▂▁▁	1.06	0.79
Cognitive impairment	2.17	0.79	1.00	1.60	2.20	2.60	5.00	▇▇▃▁▁	0.69	0.81
Emotional impairment	1.91	0.82	1.00	1.20	1.60	2.40	5.00	▇▅▁▁▁	1.29	1.88
Burnout	2.29	0.69	1.04	1.78	2.22	2.74	5.00	▆▇▅▁▁	0.76	1.29
**BAT−12**										
**Brazil Total Sample (*n* = 2217)**										
Exhaustion	3.02	0.93	1.00	2.33	3.00	3.67	5.00	▂▃▇▃▂	0.07	−0.35
Mental distance	2.07	0.92	1.00	1.33	2.00	2.67	5.00	▇▅▃▁▁	0.84	0.19
Cognitive Impairment	2.14	0.82	1.00	1.67	2.00	2.67	5.00	▇▇▅▁▁	0.69	0.46
Emotional impairment	1.84	0.82	1.00	1.00	1.67	2.33	5.00	▇▃▂▁▁	1.21	1.54
Burnout	2.27	0.69	1.00	1.75	2.17	2.67	5.00	▅▇▃▁▁	0.72	0.78
**Brazil Female Sample (*n* = 1653)**										
Exhaustion	3.06	0.91	1.00	2.33	3.00	3.67	5.00	▂▃▇▃▂	0.06	−0.35
Mental distance	2.04	0.91	1.00	1.33	2.00	2.67	5.00	▇▃▃▁▁	0.86	0.16
Cognitive impairment	2.15	0.81	1.00	1.67	2.00	2.67	5.00	▇▇▆▁▁	0.64	0.35
Emotional impairment	1.86	0.81	1.00	1.33	1.67	2.33	5.00	▇▅▂▁▁	1.15	1.33
Burnout	2.28	0.69	1.00	1.75	2.17	2.67	5.00	▅▇▃▁▁	0.70	0.55
**Brazil Male Sample (*n* = 558)**										
Exhaustion	2.91	0.94	1.00	2.33	3.00	3.67	5	▂▅▇▃▂	0.11	−0.34
Mental distance	2.14	0.93	1.00	1.33	2.00	2.67	5	▇▅▅▁▁	0.81	0.36
Cognitive impairment	2.10	0.84	1.00	1.33	2.00	2.67	5	▇▇▅▁▁	0.84	0.78
Emotional impairment	1.77	0.84	1.00	1.00	1.67	2.00	5	▇▃▂▁▁	1.42	2.24
Burnout	2.23	0.69	1.00	1.67	2.17	2.67	5	▅▇▃▁▁	0.80	1.52

**Table 7:** BAT’s raw mean scores distributional properties (workers from Portugal).

BAT-23										
Portugal Total Sample (*n* = 886)										
Dimension	*M*	*SD*	*Min*	*P*_25_	*Mdn*	*P*_75_	*Max*	Histogram	*sk*	*ku*
Exhaustion	2.91	0.74	1.00	2.38	2.88	3.38	5.00	▁▅▇▃▁	0.30	0.26
Mental distance	2.14	0.80	1.00	1.40	2.00	2.60	5.00	▇▆▃▁▁	0.70	0.09
Cognitive impairment	2.41	0.72	1.00	2.00	2.40	2.80	5.00	▃▇▅▁▁	0.41	0.54
Emotional impairment	2.08	0.70	1.00	1.60	2.00	2.40	5.00	▇▇▃▁▁	0.70	0.80
Burnout	2.45	0.62	1.00	2.04	2.39	2.83	5.00	▂▇▅▁▁	0.54	0.56
**Portugal Female Sample (*n* = 537)**										
Exhaustion	2.95	0.76	1.12	2.50	2.88	3.38	5.00	▂▇▇▅▂	0.31	0.05
Mental distance	2.10	0.79	1.00	1.40	2.00	2.60	5.00	▇▆▃▁▁	0.68	-0.04
Cognitive impairment	2.39	0.72	1.00	2.00	2.40	2.80	5.00	▃▇▅▁▁	0.33	0.51
Emotional impairment	2.08	0.69	1.00	1.60	2.00	2.40	5.00	▇▇▂▁▁	0.76	1.00
Burnout	2.45	0.63	1.04	2.00	2.39	2.83	5.00	▂▇▅▁▁	0.50	0.41
**Portugal Male Sample (*n* = 204)**										
Exhaustion	2.85	0.73	1.00	2.38	2.75	3.38	5.00	▁▆▇▃▁	0.30	0.37
Mental distance	2.25	0.85	1.00	1.60	2.20	2.80	5.00	▇▇▃▁▁	0.78	0.35
Cognitive impairment	2.49	0.76	1.00	2.00	2.40	2.85	5.00	▃▇▃▂▁	0.57	0.55
Emotional impairment	2.09	0.77	1.00	1.40	2.00	2.60	5.00	▇▇▃▁▁	0.72	0.68
Burnout	2.48	0.66	1.00	2.00	2.39	2.84	4.91	▂▇▅▂▁	0.70	0.77
**BAT-12**										
**Portugal Total Sample (*n* = 886)**										
Exhaustion	3.05	0.80	1.00	2.67	3.00	3.67	5.00	▁▃▇▃▁	0.12	0.05
Mental distance	2.27	0.78	1.00	1.67	2.00	2.67	5.00	▇▇▆▂▁	0.59	0.11
Cognitive Impairment	2.38	0.76	1.00	2.00	2.33	2.67	5.00	▅▇▇▁▁	0.48	0.59
Emotional impairment	2.02	0.71	1.00	1.33	2.00	2.33	5.00	▇▇▃▁▁	0.71	0.70
Burnout	2.43	0.61	1.00	2.00	2.33	2.75	5.00	▂▇▃▁▁	0.52	0.65
**Portugal Female Sample (*n* = 537)**										
Exhaustion	3.09	0.82	1.00	2.67	3.00	3.67	5.00	▁▃▇▃▂	0.12	-0.01
Mental distance	2.24	0.77	1.00	1.67	2.00	2.67	5.00	▇▇▇▁▁	0.55	-0.01
Cognitive impairment	2.37	0.75	1.00	2.00	2.33	2.67	5.00	▅▇▇▁▁	0.42	0.56
Emotional impairment	2.02	0.70	1.00	1.33	2.00	2.33	5.00	▇▇▃▁▁	0.75	0.83
Burnout	2.43	0.61	1.00	2.00	2.33	2.83	5.00	▂▇▅▁▁	0.46	0.53
**Portugal Male Sample (*n* = 204)**										
Exhaustion	2.96	0.80	1.00	2.33	3.00	3.33	5.00	▁▅▇▃▁	0.18	0.02
Mental distance	2.37	0.83	1.00	1.67	2.33	3.00	5.00	▆▇▆▂▁	0.65	0.21
Cognitive impairment	2.43	0.81	1.00	2.00	2.33	2.75	5.00	▃▇▆▂▁	0.76	0.72
Emotional impairment	2.02	0.77	1.00	1.33	2.00	2.33	5.00	▇▆▃▁▁	0.78	0.75
Burnout	2.44	0.66	1.00	2.00	2.33	2.83	4.92	▂▇▅▂▁	0.74	0.83

**Table 8:** Measurement model standardized latent correlations.

BAT-23						
	Burnout	Work Engagement	Negative Change	Work Overload	Role Clarity	Co-Workers Support
Burnout		−0.81 ***	0.52 ***	0.59 ***	−0.62 ***	−0.45 ***
Work Engagement	−0.75 ***		−0.61 ***	−0.32 ***	0.66 ***	0.41 ***
Negative Change	0.54 ***	−0.57 ***		0.36 ***	−0.56 ***	−0.28 ***
Work Overload	0.35 ***	0.08 *	0.16 ***		−0.38 ***	−0.21 ***
Role Clarity	−0.42 ***	0.49 ***	−0.55 ***	0.02 *^ns^*		0.47 ***
Co-workers Support	−0.45 ***	0.49 ***	−0.52 ***	−0.12 ***	0.53 ***	
**BAT-12**						
	**Burnout**	**Work Engagement**	**Negative Change**	**Work Overload**	**Role Clarity**	**Co-Workers Support**
Burnout		−0.80 ***	0.53 ***	0.61 ***	−0.62 ***	−0.44 ***
Work Engagement	−0.73 ***		−0.61 ***	−0.31 ***	0.66 ***	0.41 ***
Negative Change	0.54 ***	−0.57 ***		0.36 ***	−0.56 ***	−0.28 ***
Work Overload	0.35 ***	0.09 **	0.15 ***		−0.38 ***	−0.21 ***
Role Clarity	−0.44 ***	0.48 ***	−0.56 ***	0.02 *^ns^*		0.47 ***
Co-workers Support	−0.46 ***	0.49 ***	−0.52 ***	−0.12 ***	0.53 ***	

Note. The upper triangle values are relative to the sample from Brazil, while the lower triangle values are relative to the sample from Portugal. *^ns^ p* > 0.05; * *p* ≤ 0.05; ** *p* ≤ 0.01; *** *p* < 0.001.

## Data Availability

The data presented in this study are available on reasonable request from the corresponding author.
